# Swertianlarin, an Herbal Agent Derived from *Swertia mussotii* Franch, Attenuates Liver Injury, Inflammation, and Cholestasis in Common Bile Duct-Ligated Rats

**DOI:** 10.1155/2015/948376

**Published:** 2015-07-27

**Authors:** Liangjun Zhang, Ying Cheng, Xiaohuang Du, Sheng Chen, Xinchan Feng, Yu Gao, Shaoxue Li, Li Liu, Mei Yang, Lei Chen, Zhihong Peng, Yong Yang, Weizao Luo, Rongquan Wang, Wensheng Chen, Jin Chai

**Affiliations:** ^1^Department of Gastroenterology, Southwest Hospital, Third Military Medical University, Chongqing 400038, China; ^2^Department of Traditional Chinese Medicine, Southwest Hospital, Third Military Medical University, Chongqing 400038, China; ^3^Department of Pediatrics, Southwest Hospital, Third Military Medical University, Chongqing 400038, China; ^4^Chongqing Academy of Chinese Material Medicine, Chongqing 400065, China

## Abstract

Swertianlarin is an herbal agent abundantly distributed in *Swertia mussotii* Franch, a Chinese traditional herb used for treatment of jaundice. To study the therapeutic effect of swertianlarin on cholestasis, liver injury, serum proinflammatory cytokines, and bile salt concentrations were measured by comparing rats treated with swertianlarin 100 mg/kg/d or saline for 3, 7, or 14 days after bile duct ligation (BDL). Serum alanine aminotransferase (ATL) and aspartate aminotransferase (AST) levels were significantly decreased in BDL rats treated with swertianlarin for 14 days (*P* < 0.05). The reduced liver injury in BDL rats by swertianlarin treatment for 14 days was further confirmed by liver histopathology. Levels of serum tumor necrosis factor alpha (TNF*α*) were decreased by swertianlarin in BDL rats for 3 and 7 days (*P* < 0.05). Moreover, reductions in serum interleukins IL-1*β* and IL-6 levels were also observed in BDL rats treated with swertianlarin (*P* < 0.05). In addition, most of serum toxic bile salt concentrations (e.g., chenodeoxycholic acid (CDCA) and deoxycholic acid (DCA)) in cholestatic rats were decreased by swertianlarin (*P* < 0.05). In conclusion, the data suggest that swertianlarin derived from *Swertia mussotii* Franch attenuates liver injury, inflammation, and cholestasis in bile duct-ligated rats.

## 1. Introduction

Accumulation of toxic bile acids in hepatocytes and the amplification of inflammation in cholestasis are the major factors resulting in liver injury and fibrosis [1–3]. Multiple pathologies, that is, gallstone obstruction of the bile duct, biliary atresia, pancreas tumors, and drug toxicity, can cause persisting cholestasis which can lead to liver failure, fibrosis, cirrhosis, and death [4–6]. Ursodeoxycholic acid (UDCA) and its analogs can inhibit inflammation and enhance elimination of toxic bile acids. It is the only approved drug that is widely clinically used to treat cholestatic patients such as primary biliary cirrhosis (PBC) [7, 8]. However, one-third to two-thirds of cholestatic patients with PBC do not completely respond to UDCA [9, 10]. INT747, an FXR agonist, is a Phase II studies drug that exerts anticholestatic effects by altering bile acids metabolism in experiment models [11–14]. Therefore, the search for potential drugs that target inflammation and bile acids pools for the treatment of cholestasis is important.

Swertianlarin is an iridoid compound that is present in* Swertia davidi* Franch and* Swertia mussotii* Franch [15–18]. These two types of Swertia Franch are the traditional herbs used for the treatment of jaundice caused by viral hepatitis. It has been used for many centuries in southwest China and is supposed to protect the liver from injury [15, 18], have anti-inflammatory properties, and ameliorate cholestasis. Swertianlarin from* Enicostemma axillare* is known to have antioxidant and hepatoprotective effects against D-galactosamine-induced acute liver damage in rats [19]. Previous studies have also reported that swertianlarin inhibits inflammation in adjuvant-induced arthritis and in interleukin IL-1*β*-induced rat fibroblast-like synoviocytes [20–23]. However, the anti-inflammation effect of swertianlarin on cholestasis remains to be clarified. Furthermore, whether swertianlarin alters the bile acid pool and reduces liver injury on cholestasis is unclear.

To address whether swertianlarin derived from* Swertia mussotii* Franch attenuates inflammation and liver injury on cholestasis, we assessed the liver injury, serum proinflammatory cytokine levels, and the concentrations of serum bile acids in a bile duct-ligated rat model treated with swertianlarin.

## 2. Materials and Methods

### 2.1. Chemicals

Swertianlarin, isolated from* Swertia mussotii* Franch as reported previously [24], was kindly provided by Chongqing Academy of Chinese Material Medical with a purity of 98% as analyzed by HPLC. All other chemicals were of analytical grade and purchased from Sigma-Aldrich (Sigma-Aldrich Chemical Co., St. Louis, MO, USA).

### 2.2. Animals and Treatments

The male Sprague-Dawley (SD) rats were provided by the Center of Laboratory Animals of Third Military Medical University, Chongqing, China. All experimental protocols were approved by the Ethics Committee of Third Military Medical University. Efforts were made to reduce animal suffering and minimize animal number used. Animals were housed in plastic cages with free access to food and water under a 12 h light/dark cycle. The normal rats were given swertianlarin in 4 groups: 0, 10, 50, and 100 mg/(kg/d), dissolved in 1% Tween-20 saline, *n* = 7, by gavage for 14 days. All rats survived and the serum biochemistries alanine aminotransferase (ALT), aspartate aminotransferase (AST), alkaline phosphatase (ALP), total bile acids (TBA), total bilirubin (TBIL), and direct bilirubin (DBIL) were normal in the study (data not shown). In this study, rats were randomly divided into three groups (*n* = 7 per group): sham saline, BDL without swertianlarin, and BDL with swertianlarin with each containing from five to seven animals. In the BDL swertianlarin group, rats were pretreated with swertianlarin 100 mg/(kg/d) dissolved in 1% Tween-20 by gavage for 1 d and then underwent BDL followed by continued administration of swertianlarin for 3, 7, and 14 d. In the BDL saline groups, rats underwent BDL after pretreatment with only 1% Tween-20 saline by gavage for 1 d and then continued treatment with only 1% Tween-20 saline for 3, 7, and 14 d. In the sham saline control group, rats were pretreated with 1% Tween-20 saline by gavage for 1 d and underwent a sham-operation followed by only 1% Tween-20 saline for 3, 7, and 14 d. After 3, 7, and 14 d, animals were sacrificed randomly between 9:00 am and 11:00 am. Blood was placed on ice for 60 min and centrifuged (8000 g, 10 min) to prepare serum. The serum was immediately stored at −80°C until used. The liver samples were immediately cut into small pieces and frozen in liquid nitrogen until used. The collected serum and liver samples were used for biochemistry and histopathology studies as described previously [25].

### 2.3. Serum Biochemistry

The concentrations of alanine aminotransferase (ALT), aspartate aminotransferase (AST), alkaline phosphatase (ALP), total bile acids (TBA), total bilirubin (TBIL), and direct bilirubin (DBIL) in serum samples were analyzed by standard enzymatic assays using commercial kits.

### 2.4. Determination of Serum Proinflammatory Cytokines TNF*α*, Interleukin-1*β*, and Interleukin-6

Serum samples from sham operated rats and bile duct-ligated rats with and without swertianlarin were collected before sacrifice (*n* = 7 per each group) and stored at −80°C until analysis. Serum cytokines, tumor necrosis factor alpha (TNF*α*), interleukin-1 beta (IL-1*β*), and IL-6 levels were determined by ELISA Kits (BlueGene Biotechnology, Shanghai, China) according to the manufacturer's instructions [25, 26].

### 2.5. Serum Bile Salts and Lipids Determination

The concentrations of serum bile acids chenodeoxycholic acid (CDCA), taurochenodeoxycholic acid (TCDCA), cholic acid (CA), taurocholic acid (TCA), deoxycholic acid (DCA), taurodeoxycholic acid A (TDC), tauroursodeoxycholic acid (TUDCA), tauro-alpha/beta-muricholic acid (T*α*/*β*MCA), alpha-muricholic acid (*α*MCA), and beta-muricholic acid (*β*MCA) levels, serum lipid triglyceride hydrolase (Tgh), triglyceride (TG), high-density lipoprotein cholesterol (HDL-C), and low-density lipoprotein cholesterol (LDL-C) were measured by standard enzymatic assays using commercial kits purchased from BlueGene Biotech (Shanghai, China), according to the manufacturer's protocol as described previously [27, 28].

### 2.6. Histopathology

Rat liver samples were fixed in 4% formalin and subjected to standard histological procedures and paraffin embedding. Liver sections (5 *μ*m in thickness) were stained with hematoxylin and eosin and evaluated for histological lesions.

### 2.7. Statistical Analysis

All values were expressed as mean ± standard deviation (SD). One-way analysis of variance followed by Dunnett's multiple comparison post hoc test was used for statistical analysis of data using GraphPad Prism 6.01 (GraphPad Software Inc., San Diego, CA). A value of *P* < 0.05 was considered to be statistically significant.

## 3. Results

### 3.1. Swertianlarin Attenuates Liver Injury in Common Bile Duct-Ligated Rats

**Figure 1:**
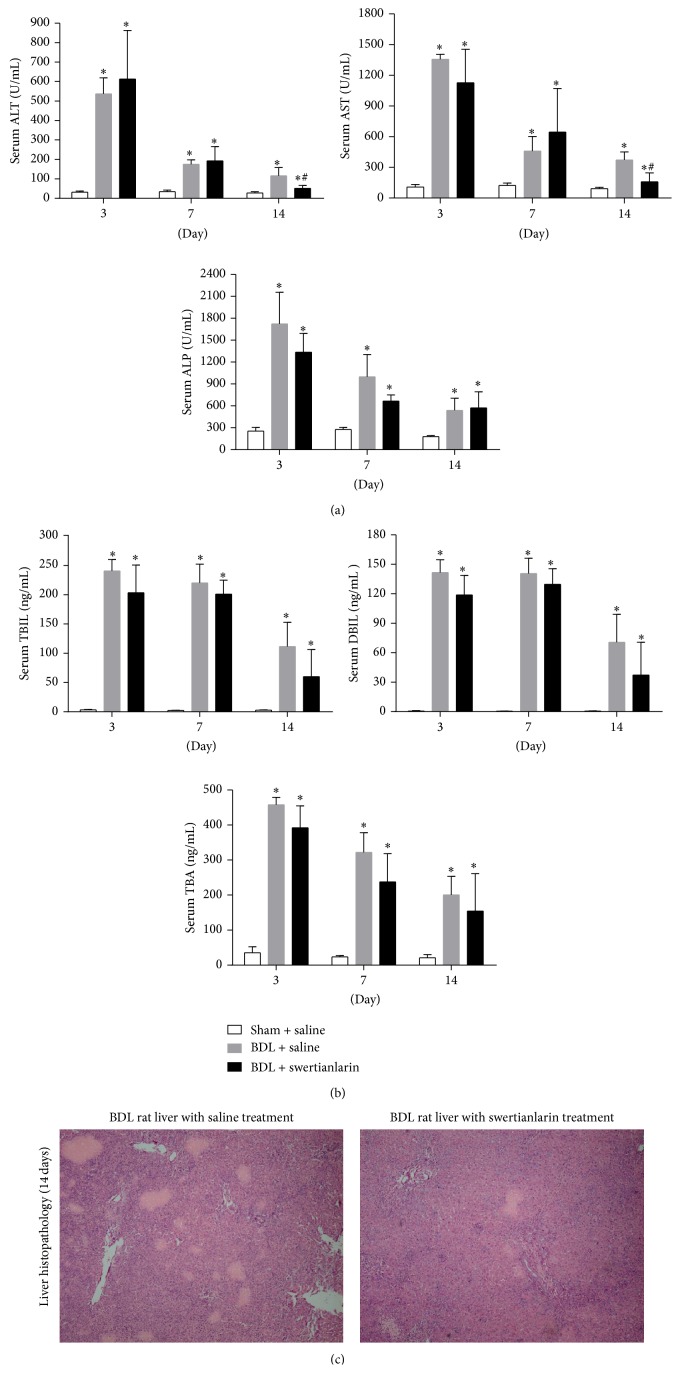
Changes of serum liver injury biomarkers and histopathology in swertianlarin-treated BDL rats. (a) Changes in ALT, AST, and ALP levels in sham rats with saline, BDL rats without swertianlarin, and BDL rats with swertianlarin for 3, 7, and 14 d. (b) Alterations of biomarkers TBIL, DBIL, and TBA levels in sham rats with saline, BDL rats without swertianlarin, and BDL rats with swertianlarin for 3, 7, and 14 d. (c) Liver histopathology (4X) in BDL rats with or without swertianlarin for 14 d. Data were analyzed using one-way analysis of variance followed by Dunnett's multiple comparison post hoc test. ^*^ *P* < 0.05 versus sham group with saline; ^#^ *P* < 0.05, versus BDL group with saline. *n* = 7 per group. Saline, 1% Tween-20 saline; swertianlarin dissolved in 1% Tween-20 saline.

Figure 1(a) shows that serum ALT, AST, and ALP were significantly increased in both BDL rats treated with and those without swertianlarin for 3, 7, and 14 days, compared to sham group with saline (*P* < 0.05, Figure 1(a)). However, serum ALT and AST levels for 14 d were markedly decreased in the BDL rats with swertianlarin (54% and 52% of saline BDL group, resp.; *P* < 0.05). However, the levels of these two markers were not significantly different at the earlier time points of 3 and 7 d, compared to the BDL rats without swertianlarin (Figure 1(a)). Furthermore, the serum ALP levels for 3, 7, and 14 d were not significantly different in both BDL with and without swertianlarin groups at any time (Figure 1(a)). The reduced liver injury in BDL rats with swertianlarin for 14 d was further confirmed by liver histopathology (Figure 1(c)). Moreover, the serum TBIL, DBIL, and TBA levels were noticeably increased in all BDL groups. There were no significant differences in TBIL, DBIL, and TBA between BDL groups with or without swertianlarin (Figure 1(b)). Nevertheless, the serum levels of TBIL and DBIL tended to be lower in the BDL group with swertianlarin compared to the BDL group without swertianlarin for 14 d (Figure 1(b)). These results indicated that swertianlarin treatment decreased liver injury, whereas the serum TBIL and TBA levels in BDL rats remained unaltered for 14 d.

### 3.2. Swertianlarin Has Anti-Inflammatory Effect on BDL Rats

**Figure 2:**
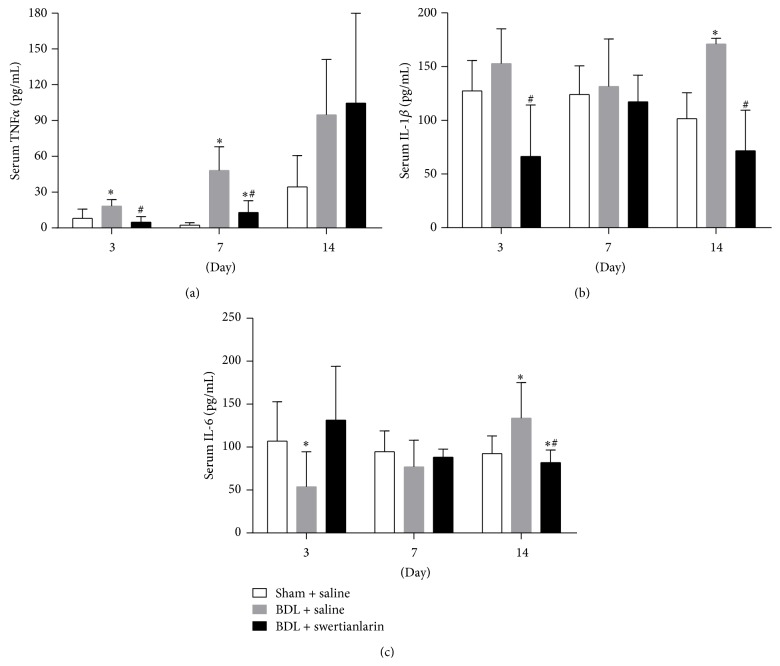
Alterations of serum proinflammatory cytokines TNF*α*, IL-1*β*, and IL-6 levels by swertianlarin in BDL rats. (a) The changes of serum TNF*α* in the sham operated, BDL with swertianlarin, and BDL without swertianlarin groups for 3, 7, and 14 d. (b) The alterations of serum IL-1*β* in sham operated, BDL with swertianlarin, and BDL without swertianlarin groups for 3, 7, and 14 d. (c) The determination of serum IL-6 in sham operated rats and BDL rats treated with or without swertianlarin after 3, 7, and 14 d. Data were analyzed as described in Materials and Methods. ^*^ *P* < 0.05 versus sham group with saline; ^#^ *P* < 0.05, versus BDL group with saline. *n* = 7 per group. Saline, 1% Tween-20 saline; swertianlarin dissolved in 1% Tween-20 saline.

**Figure 3:**
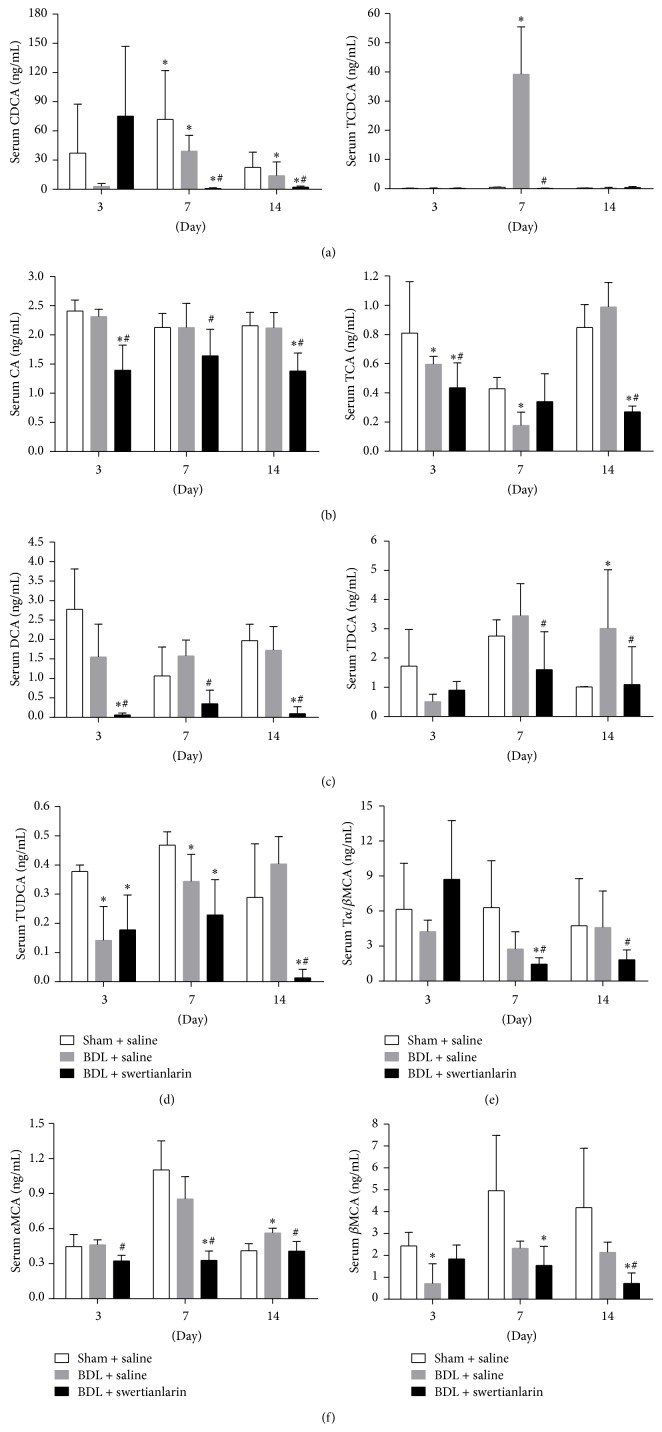
Alterations of serum bile salts by swertianlarin in BDL rats. (a) The concentrations of serum CDCA and TCDCA in sham operated rats and BDL rats with or without swertianlarin for 3, 7, and 14 d. (b) The changes in serum bile salts CA and TCA in sham operated, BDL with swertianlarin, and BDL without swertianlarin groups for 3, 7, and 14 d. (c) The determination of serum DCA and TDCA in sham operated rats and BDL rats treated with or without swertianlarin after 3, 7, and 14 d. (d) Serum TUDCA, (e) T*α*/*β*MCA, and (f) *α*MCA and *β*MCA were measured in sham operated group, BDL with swertianlarin group, and BDL without swertianlarin group for 3, 7, and 14 d. Data were analyzed as described in Materials and Methods. ^*^ *P* < 0.05 versus sham group with saline; ^#^ *P* < 0.05, versus BDL group with saline. *n* = 7 per group. Saline, 1% Tween-20 saline; swertianlarin dissolved in 1% Tween-20 saline.

To investigate whether swertianlarin has anti-inflammatory effects, the concentration of serum proinflammatory cytokines TNF*α*, IL-1*β*, and IL-6 were measured by ELISA assay in the sham operated group and BDL groups with or without swertianlarin. The results showed that the serum TNF*α* levels for 3 and 7 d were dramatically increased while they were not significantly increased for 14 d in cholestatic rats without swertianlarin compared to sham operated rats (Figure 2(a)). However, the levels of serum TNF*α* in cholestatic rats with BDL for 3 and 7 d were significantly lower with swertianlarin treatment, 63% and 64% of saline BDL group, respectively (*P* < 0.05). However, they were not significantly different for 14 d, compared to the BDL group without swertianlarin (Figure 2(a)). Furthermore, serum IL-1*β* levels were significantly increased for 14 d while they were unchanged for 3 and 7 d in the BDL group with swertianlarin compared to those of the sham operated group (Figure 2(b)). However, the serum IL-1*β* levels for 3 and 14 d were noticeably lower in the swertianlarin BDL group (43% and 42% of BDL group without swertianlarin, resp.; *P* < 0.05). Moreover, the serum IL-1*β* levels were not altered by swertianlarin for 7 d (Figure 2(b)).  Figure 2(c) shows that serum IL-6 levels were increased for 14 d but was decreased for 3 d (*P* < 0.05) and was not different for 7 d in BDL group without swertianlarin compared to the sham operated group (Figure 3(c)). Serum IL-6 in the swertianlarin BDL group for 14 d was 61% of saline BDL group (*P* < 0.05), whereas the serum IL-6 levels were not affected by swertianlarin for 3 and 7 d (Figure 3(c)). These results demonstrated that swertianlarin treatment reduced levels of serum proinflammatory cytokines TNF*α*, IL-1*β*, and IL-6 in this rat model.

### 3.3. Swertianlarin Alters the Concentration of Serum Bile Salts in Rats with Bile Duct Ligation

The accumulation of bile salts is the major factor resulting in the liver injury in cholestasis [1, 3, 21]. Because an apparent decrease in liver injury in cholestatic rats was associated with swertianlarin treatment, it was determined if swertianlarin alters the concentration of serum bile salts in cholestasis. The results showed that CDCA was lower in BDL rats without swertianlarin for 3, 7, and 14 days compared with sham operated rats (*P* = 0.31, *P* < 0.05, and *P* < 0.05, Figure 3(a)). In contrast, the TCDCA levels were increased in the BDL rats without swertianlarin for 7 d but were unaltered after 3 and 14 d (Figure 3(a)). The administration of swertianlarin decreased CDCA levels after 7 and 14 d and reduced TCDCA levels after 7 d in BDL rats (*P* < 0.05, Figure 3(a)). The concentration of serum CA was unchanged in BDL rats without swertianlarin, compared to the sham operated group. However, the serum CA levels were decreased in BDL rats with swertianlarin for 3 (*P* < 0.05), 7, and 14 d (*P* < 0.05, Figure 3(b)). Moreover, TCA levels were only decreased in BDL rats without swertianlarin after 3 and 7 d (*P* < 0.05, Figure 3(b)), but not significantly changed after 14 d. However, serum TCA was significantly lower in BDL rats with swertianlarin after 3 and 14 d (*P* < 0.05, Figure 3(b)), but not after 7 d, when compared to BDL rats without swertianlarin. Figure 3(c) (left) shows that serum DCA levels were similar in BDL rats without swertianlarin, compared with sham group. However, the serum DCA levels were decreased in BDL rats with swertianlarin for 3, 7, and 14 d (*P* < 0.05 for all, Figure 3(c) left). In contrast, serum TDCA levels were increased in BDL rats without swertianlarin for 14 d but were not elevated with swertianlarin treatment (*P* < 0.05, Figure 3(c) right). There were no difference in TDCA levels in BDL rats with or without swertianlarin after 3 d, whereas TDCA levels were lower in BDL rats with swertianlarin after 7 d, compared with BDL rats without swertianlarin (*P* < 0.05, Figure 3(c)).  Figure 3(d) showed that TUDCA levels were lower in cholestatic rats without swertianlarin after 3 and 7 d (*P* < 0.05) but were not different after 14 days. Administration of swertianlarin decreased TUDCA levels in BDL rats after 14 d (*P* < 0.05, Figure 3(d)) compared to BDL rats without swertianlarin. Furthermore, serum T*α*/*β*MCA levels were nonsignificantly different between the sham group and BDL groups without swertianlarin for 3, 7, and 14 d but were decreased in the BDL group with swertianlarin after 7 and 14 d, compared to the BDL group without swertianlarin (*P* < 0.05, Figure 3(e)).  Figure 3(f) (left) shows that serum *α*MCA levels were similar in the BDL without swertianlarin group after 3 and 7 d but were higher in the BDL group without swertianlarin compared to the sham group for 14 d (*P* < 0.05). However, serum *α*MCA levels were decreased in the BDL group with swertianlarin for 3, 7, and 14 d (*P* < 0.05), compared with the BDL group without swertianlarin (Figure 3(f)). Serum *β*MCA levels were lower in the BDL group without swertianlarin for 3 d (*P* < 0.05) while they were not significantly changed for 7 d and 14 d, compared with the sham group. Administration of swertianlarin for 14 d significantly decreased serum *β*MCA levels in the BDL rats (*P* < 0.05, Figure 3(f), right). These results indicated that swertianlarin administration was associated with lower levels of conjugated and unconjugated bile salts in BDL rats.

### 3.4. Swertianlarin Does Not Affect Lipids Levels in BDL Rats

**Figure 4:**
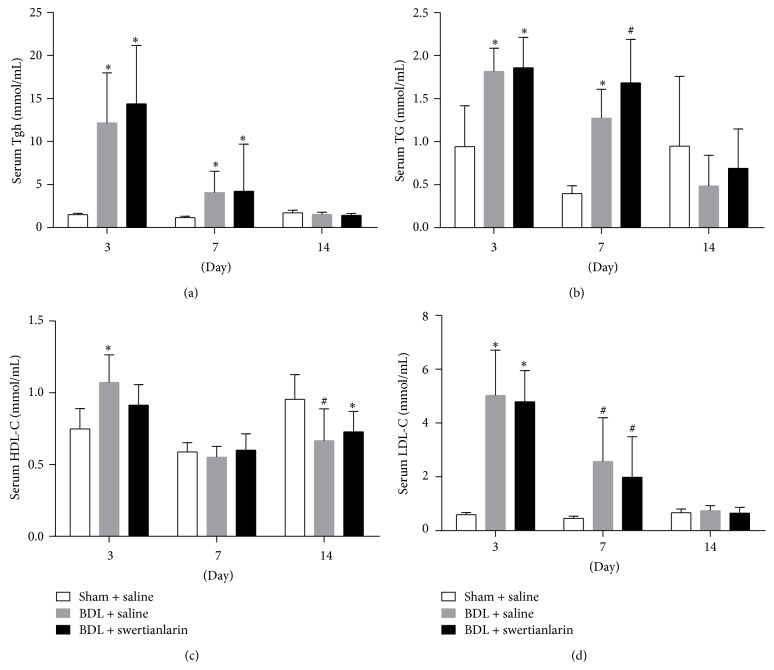
The lipids Tgh, TG, HDL-C, and LDL-C levels were unaffected by swertianlarin in bile duct-ligated rats. (a) Serum Tgh, (b) TG, (c) HDL-C, and (d) serum LDL-C levels were measured in sham operated rats, BDL rats without swertianlarin, and BDL rats with swertianlarin for 3, 7, and 14 d. Data were analyzed as described in Materials and Methods. ^*^ *P* < 0.01; ^#^ *P* < 0.05, versus sham group with saline. *n* = 7 per group. Saline, 1% Tween-20 saline; swertianlarin dissolved in 1% Tween-20 saline.

Serum Tgh, TG, and LDL-C levels for 3 and 7 d were higher, but unchanged for 14 d in BDL saline rats, compared with the sham group (*P* < 0.01, Figures 4(a), 4(b), and 4(d)). However, the HDL-C levels were increased for 3 d but decreased for 14 d in BDL rats (Figure 4(c)). Furthermore, there was no difference in serum HDL-C in BDL rats after 7 d (Figure 4(c)). The results showed that levels of Tgh, TG, HDL-C, and LDL-C were not significantly different in BDL rats with or without swertianlarin (Figure 4). These results suggest that lipid metabolism was not affected by swertianlarin in this model.

## 4. Discussion

Persisting cholestasis caused by bile duct obstruction (i.e., gallstones and pancreas tumors), hepatitis, and drugs can lead to liver failure, fibrosis, and cirrhosis [1–6]. The accumulation of bile acids in hepatocytes and hepatic chronic inflammation plays key roles in cholestatic liver injury [1–3]. The drugs that enhance elimination of toxic bile acids, such as UDCA and INT747 are beneficial for cholestatic patients [7–14]. In the present study, it was observed that swertianlarin attenuates liver injury and inflammation and is associated with lower concentrations of bile salts in BDL rats.

Models of cholestasis can be produced by administration of some drugs, for example, *α*-naphthylisothiocyanate (ANIT) (intrahepatic cholestasis), or by bile duct ligation (extrahepatic cholestasis) [27–29]. The presence of toxic bile acids is the major factor resulting in liver injury in cholestasis [1, 2]. The present study showed that certain conjugated and unconjugated bile acids in serum were reduced in BDL rats by swertianlarin treatment for 3, 7, and 14 d, implying that swertianlarin attenuates cholestasis. Serum bile acid levels were reduced in BDL rats treated with swertianlarin for 14 d. These data may explain why levels of serum markers of liver injury, ALT, and AST, as well as liver necrosis determined by histopathology, were significantly decreased in BDL rats treated with swertianlarin for 14 d. Many studies have demonstrated that inhibition of bile acid synthetic enzymes (e.g., Cyp7a1), upregulation of detoxification enzymes (e.g., Cyp3a11 and Ugt2b), and bile acids transporters (e.g., Mrp3 and Mrp4) contribute to the repression of bile acid synthesis, the reduction of bile salt toxicity. This may occur by increasing water solubility and elimination of bile acids, alleviating cholestasis and liver injury in cholestatic animal models and in human cholestatic patients using UDCA and INT747 [1, 2, 25]. The differences in timing of alterations of serum bile acids levels in BDL rats may be due to changes in hepatic bile acid transporters, synthetic and detoxification enzymes regulated by swertianlarin. However, this hypothesis needs to be tested by more in-depth studies in future. Moreover, it was found that the lipid levels Tgh, TG, HDL-C, and LDL-C were not different in swertianlarin-treated BDL rats compared to BDL rats without swertianlarin. However, serum lipid levels were severely disturbed in all of BDL rats. These results suggested that BDL not only affected bile acid homeostasis, but also interfered with lipid homeostasis. In addition, one recent study also reported that swertianlarin from* Enicostemma axillare* had antioxidant and hepatoprotective effects against D-galactosamine-induced acute liver damage in rats [19]. These support the protective role of swertianlarin on cholestasis.

We also demonstrated that elevated serum levels of proinflammatory cytokines TNF*α* for 3 and 7 d, IL-1*β*, and IL-6 for 14 d in BDL rats were significantly reduced by swertianlarin treatment, implying that swertianlarin has an anti-inflammatory effect on cholestatic process. These observations in BDL rats with swertianlarin treatment are similar to those previously described in adjuvant-induced arthritis and IL-1*β*-induced rat fibroblast-like synoviocyte models [20, 21]. A recent study reported that inflammation plays an important role in the progression of the pathology of cholestasis [3]. Knockout of TNF*α* in a cholestatic mouse model reduced liver injury and fibrosis [30]. Therefore, swertianlarin may also alter the progression of cholestasis by inhibiting inflammation. However, whether swertianlarin exerts an anti-inflammatory effect on cholestasis by the activation of NF-*κ*B and JAK signaling, as reported previously [20], remains to be clarified.

Although the swertianlarin-treated BDL rats had less liver injury, inflammation, and cholestasis than those without swertianlarin, these results are from one cholestasis model. Whether swertianlarin also exerts the protective role in other cholestatic models (e.g., lipopolysaccharide-induced cholestasis) or human cholestasis (e.g., primary biliary cirrhosis) needs further studies. Moreover, the molecular mechanism by which swertianlarin affects cholestasis remains to be determined.

## 5. Conclusions

The results demonstrated that swertianlarin from* Swertia mussotii* Franch attenuates liver injury, inflammation, and cholestasis in BDL rats. The findings of the present study contribute to understanding the protective role of swertianlarin in cholestasis and provide preliminary data for the development of potential drugs for the treatment of cholestasis.
